# Teaching Happiness to Teachers - Development and Evaluation of a Training in Subjective Well-Being

**DOI:** 10.3389/fpsyg.2019.02703

**Published:** 2019-12-03

**Authors:** Tobias Rahm, Elke Heise

**Affiliations:** Institute of Educational Psychology, Technical University of Braunschweig, Braunschweig, Germany

**Keywords:** positive psychology, positive education, subjective well-being, teachers’ well-being, teacher training, positive interventions, positive emotions

## Abstract

Teachers’ health is a persistent challenge for educational systems all over the world. Moreover, research results – especially in the domain of positive psychology – indicate that high levels of well-being are associated with additional benefits improving teachers’ professional performance. Therefore, a training to foster subjective well-being with one training day, two booster sessions, and exercises before, during, and after the meetings was developed. It consisted of about 10 h of face-to-face time and about 3 h for the exercises in total over a 5-week training period. Main contents were conditions and consequences of positive and negative emotions and well-being, emotion regulation, time management, savoring and gratitude and the application of positive psychological interventions (like *Three Good Things*). Analyses of planned contrasts by means of a waiting control group design with three measurement points (pre, post, and follow-up) showed a significantly higher increase for the training group (*n* = 42) than for the control group (*n* = 47) in the frequency of positive emotions, life satisfaction, and flourishing (interaction effects *d* = 0.44, *d* = 0.31, and *d* = 0.32) and a significantly stronger decrease in the frequency of negative emotions, perceived stress, and experiencing emotional exhaustion (interaction effects *d* = 0.69, *d* = 0.51, and *d* = 0.47) from pre to 1-month follow-up. Training effects were also visible up to 5 months, although no control group could be realized for this period due to the field approach.

## Introduction

The overall aim of our educational systems is to provide students with the best possible preparation for their lives. Even if the question of the correct operationalization of this goal and the priorities and methods to be chosen accordingly is answered differently in different societies, one constant remains: for the best possible education, those who deliver it must be able to make the best possible use of their potential. For several decades, the scientific community has been researching the conditions and consequences of the relatively high levels of stress, emotional exhaustion, burnout, and health impairments among teachers (e.g., De Heus and Diekstra, 1999; Gray et al., 2017). Different aspects of the teaching profession are studied to explain these findings. Skaalvik and Skaalvik (2018) summarize that “Teacher stress is typically conceptualized as unpleasant emotions resulting from aspects of the work as a teacher” (p. 1251). Beside other stressors discussed (like student diversity, discipline problems, conflicts with colleagues, lack of administrative support, etc.), time pressure was found by Skaalvik and Skaalvik (2017) to have strong relations to emotional exhaustion, which in turn is one of the main predictive factors to develop burnout symptoms (Maslach et al., 1996).

It is clear that teachers with health problems are not able to fully exploit the potential of their abilities, which has a negative impact on their professional performance (e.g., instructional performance; Klusmann et al., 2008) and also causes considerable economical costs through medical-psychological treatment and absenteeism. Improving teachers’ health therefore remains an important goal. A new perspective on health is introduced by positive psychology, which aims at promoting holistic well-being and individual growth and at contributing to the unfolding of one’s full potential. One of the central concepts of positive psychology and the target construct of this study is subjective well-being (SWB), which is regarded as the scientific concept that comes closest to the general term “happiness” (Diener and Scollon, 2014; for an overview of well-being terms see Diener et al., 2018). SWB is composed of life satisfaction and the frequency of positive and negative affect (Diener, 1984; Pavot and Diener, 2013). All three domains of SWB are independent from each other and have distinctive associations with other variables (Diener et al., 2017). Satisfaction with life (SWL) is the cognitive component of SWB and encompasses “People’s explicit and conscious evaluations of their lives, often based on factors that the individual deems relevant” (Diener et al., 2018, p. 3). People with high SWL therefore usually have a good balance of what they desire and what they actually have in their lives – especially in persistent domains like health, income or quality of one’s work. The affective component of SWB consists of the frequency of positive affect (PA) and negative affect (NA). From a neurobiological point of view, PA is strongly connected to our reward system where it serves as a source of motivation and supports learning processes (Esch, 2012). Following the *broaden-and-build theory of positive emotions* by Fredrickson (1998, 2013), the “experiences of positive emotions broaden people’s momentary thought-action repertoires, which in turn serves to build their enduring personal resources, ranging from physical and intellectual resources to social and psychological resources” (Fredrickson, 2001, p. 218). These additional resources facilitate success, personal growth, and a “successful life” – which in turn leads to more positive emotions and to a self-reinforcing upward spiral. NA, on the other hand, occurs if needs and relevant values and goals are threatened leading to a narrowing of the thought-action repertoires toward fight or flight responses (e.g., Garland et al., 2010) initiated through the release of stress hormones (Costandi, 2015). NA is not negative *per se* as it yields fast information on situations, helps to avoid threats to people’s goals (e.g., Garland et al., 2010), but experiencing too much negativity “leads to health problems in the long run” (Diener et al., 2017, p. 2). In addition to these direct effects of SWL, PA, and NA, research also shows favorable outcomes of high overall SWB. In an extensive meta-analysis, Lyubomirsky et al. (2005) examined 225 empirical studies that tested the effects of SWB (or individual components thereof) on different areas of life. The findings show, among other things, that people with a high level of well-being live longer (Danner et al., 2001; Veenhoven, 2008), have a more efficient immune system (Barak, 2006), are more efficient and successful at work (Achor, 2010), are more creative (Baas et al., 2008), and have more versatile social relationships (Rodríguez-Pose and von Berlepsch, 2012). High SWB is therefore not only highly relevant for people’s physical and psychological health but also for their personal growth and their performance in work and life.

### Teachers’ Well-Being Matters

The internationally booming field of positive education applies findings from positive psychology to schools and, in addition to academic achievements, focuses on the goal of promoting the holistic well-being of everyone involved in school (e.g., Norrish, 2015; Larsen, 2016). The most important actors in achieving school objectives are teachers (Hattie, 2003). If they are to deliver education with high quality professional performance it would be favorable to secure a good state of health and even more a high level of SWB for them. While Lyubomirsky and Lepper (1999) conclude that high SWB acts as a protective factor against depression and burn-out, Sutton and Wheatley (2003) emphasize in their review, that, for example, positive and negative emotions of teachers significantly influence student outcomes. Of particular importance for the classroom is the effect of “emotional contagion,” the transfer of frequent positive emotions from teachers to their students (Frenzel et al., 2009). The frequency of positive emotions also corresponds to frequent use of effective teaching strategies (Moè et al., 2010). Specifically, Kunter (2013) was able to show that affective well-being (here enthusiasm for teaching) of mathematics teachers explains changes in mathematical achievement and the students’ enjoyment of mathematics. In addition, positive affect is connected with intrinsic motivation and the ideal image of the “passionate teacher,” who is particularly good at stimulating his or her students and motivating them to develop their potential to the greatest possible extent (Kunter and Holzberger, 2014). More recently, Buonomo et al. (2019) found that experiencing positive emotions toward students partially mediates the negative influence of negative emotions toward students on teachers’ self-efficacy, which in turn is an important predictor for professional performance of teachers and a protective factor against teachers’ ill-being (Zee and Koomen, 2016).

The findings thus provide clear evidence that SWB is linked to many desirable outcomes that are also highly relevant for the education system and the teaching profession. According to general and teacher-specific findings, increasing SWB of teachers should contribute to improving their quality of health and have a positive impact on the teaching-learning processes in schools (see also Gray et al., 2017).

### Training of Well-Being

The question now arising is whether SWB can be improved. In fact, at the end of the 1990s, it was still assumed that an individual set-point existed for SWB, to which it would always return in the long term. Lykken and Tellegen (1996) came to the conclusion that “it may be that trying to be happier is as futile as trying to be taller” (p. 189). Recent research has shown, however, that this assumption was not true. In their remarkable review article, Luhmann and Intelisano (2018) examine studies on hedonic adaptation and come to the conclusion that “together, these studies indicate that SWB is relatively stable but nonetheless changeable” (p. 13). In the following, we describe different psychological interventions or programs aiming at the enhancement of PA and SWL – so called positive psychological interventions (PPIs) – and at the reduction of negative emotions.

#### Positive Psychological Interventions

With the introduction of positive psychology at the turn of the milennium, more and more studies were carried out with the explicit aim of achieving a sustainable increase in well-being. For this purpose, PPIs (cf. Parks and Biswas-Diener, 2013) were developed and evaluated in various settings. One of the first large randomized placebo-control trials on PPIs was conducted by Seligman et al. (2005). Of the total of five PPIs used in this study, the exercise *Three Good Things* stood out in particular causing medium to large long-term effects: participants were asked to write down three things that went well each day and to provide a causal explanation for each good thing. The PPI was instructed completely online and should be performed for only 1 week. Participants were able to significantly increase their well-being and decrease their depressive symptoms in comparison to the placebo control group, where participants had to write about early memories every night for 1 week. The effect remained significant at a 6-months follow-up and could be replicated several times (Mongrain and Anselmo-Matthews, 2012; Gander et al., 2013). Several meta-analyses (Sin and Lyubomirsky, 2009; Bolier et al., 2013) support the finding that SWB (or components thereof) can be sustainably increased by PPIs. However, studies on individual online interventions predominate so far – probably because they are easier to implement and have the advantage of wider dissemination.

Other publications address more complex intervention programs with multiple exercises and background information on conditions and consequences of well-being. A good example is provided by Feicht et al. (2013), who evaluated a 7-week “happiness training” conducted online. The main focus was on informative video clips and texts, various PPIs (e.g., *Three Good Things* and *Gratitude Letter*, see below), and reflection exercises on personal behavior and experience. Questionnaire data show large effects, e.g., for different measures of well-being and stress reduction between training and waiting control group 4 weeks after completion of training.

Presence-based trainings offer the advantages of more direct interchange and sharing reflections and might better reach people who could benefit from a training but would not participate in an online training. University seminars in positive psychology represent a distinct group of presence-based complex interventions, which are usually offered weekly over the course of one semester. In addition to teaching the theoretical contents of positive psychology, part of the workload consists of getting to know PPIs through self-application. Goodmon et al. (2016) report significant improvements in various dimensions of well-being (including general happiness, SWL, perceived stress, and depression) in a pre–post design. Participants of a parallel seminar in social psychology served as a control group. Another group of face-to-face multi-component interventions is positive psychotherapy (Seligman et al., 2006). Positive psychotherapy is mainly applied by psychotherapists in clinical settings, containing elements of information and reflection in groups and PPIs as homework between sessions. In a current study in a clinical setting with predominantly depressive participants, Furchtlehner et al. (2019), for example, found better results after 14 group sessions of positive psychotherapy than after conventional cognitive-behavioral therapy.

Basically, it can be assumed that PPIs or more complex programs can improve well-being in the long term by changing the participants’ individual experience and behavior. Hendricks et al. (2019) conducted a meta-analysis of multi-component PPIs and conclude that they have “a small effect on SWB and depression, and a small to moderate effect on psychological well-being” (p. 1). None of the 50 included studies, however, aimed at the training of teachers. Although more and more programs for positive education aiming at the fostering of students’ well-being are being developed internationally (see Norrish, 2015; Larsen, 2016) and most of them contain teacher training in positive psychology, the evaluation of trainings to improve teachers’ SWB has so far been neglected. In addition, the aim of increasing teachers’ well-being could be well-served by combining PPIs (targeting PA and SWL) with interventions specifically aiming at the reduction of NA.

#### Psychological Interventions on Stress and Negative Emotions

As pointed out above, dealing with stress and NA is a persistent challenge in teachers’ occupational life and a threat to various dimensions of health. In the field of emotion regulation, processes to influence occurrence, experience, and expression of emotions are studied. Gross (1998) summarizes five points to regulate emotions: “(a) selection of the situation, (b) modification of the situation, (c) deployment of attention, (d) change of cognitions, and (e) modulation of responses” (Gross, 1998, p. 271). Various intervention programs target different strategies to improve emotion regulation processes, leading to various positive outcomes (for a review see for example Gratz et al., 2015). Elaborated clinical emotion regulation programs like the *affect regulation training* (ART, Berking and Whitley, 2014) use for example psychoeducation, muscle relaxation, breathing relaxation, non-judgmental perception of emotions, acceptance and tolerance of emotions, compassionate self-support, identification of the causes of one’s emotional response and active modification of emotions. ART could show its effectiveness in various applications like for example in the reduction of depressive symptoms (e.g., Berking et al., 2013). A special aspect of stress reduction interventions are time management trainings. As time pressure is one of the main stressors leading to teachers’ experience of distress (Skaalvik and Skaalvik, 2018), time management interventions are quite often delivered in in-school trainings in Germany. Claessens et al. (2007) reviewed 32 empirical studies and defined time management as “behaviors that aim at achieving an effective use of time while performing certain goal-directed activities” (p. 262). Examples for such behaviors are setting goals, planning tasks, prioritizing activities, striving for self-awareness of one’s time use, knowing the limit of one’s capabilities etc. The authors concluded that “time management behaviors relate positively to perceived control of time, job satisfaction, and health, and negatively to stress” (p. 255).

### Aims of the Present Training

Altogether, research results show that high levels of SWB are beneficial in various areas of life and especially in the teaching profession, and that SWL and the frequency of PA and NA are improvable through (multi-component) PPIs and other psychological interventions like trainings in emotion regulation or time management.

The main aim of the present training therefore is to influence the individual behavior and experience of the participants in such a way that (1) positive emotions are experienced more frequently, (2) negative emotions are experienced less frequently, and (3) satisfaction with one’s own life is increased. The methods to achieve these goals are positive psychoeducation (information), exercises, and homework in the sense of cognitive-behavioral therapy as well as reflections on experience and behavior in everyday life and on the experiences gained during the exercises.

Another central concept in positive psychology is flourishing, which can be understood as a more comprehensive construct of well-being describing a state of optimal social and psychological functioning (e.g., Su et al., 2014). As PPIs generally target the promotion of flourishing, we also included this construct in the development and evaluation of the training. Additionally, we were interested whether we could also stimulate and find changes in self-efficacy (Schwarzer and Jerusalem, 1995) and attributional style (Weiner, 2000), which are both variables that could explain the surprisingly high impact of the PPI *Three Good Things* (which is a core PPI in the training, see below) on well-being.

In order to create an attractive offer for school-internal and cross-school further education, the training should require as little effort as possible for the participating teachers and be easy to integrate into everyday school life.

### Hypotheses

We postulate that the training described below contributes to the improvement of different components of SWB (SWL, PA, and NA), to flourishing, and to emotional exhaustion and perceived stress (as additional measures of NA). In addition to the main aim of increasing SWB, we assume that it might also improve self-efficacy beliefs and attributional styles. Both constructs are not common target variables in (multi-component) PPIs, but as the conception of the training also includes contents that might evoke changes regarding these constructs, we enclosed them as additional hypotheses.

Compared to the waiting control group, the training group reports:

• a higher increase of the frequency of PA (H1),
• a higher increase of SWL (H2),
• a higher increase of flourishing (H3),
• a stronger decrease of the frequency of NA (H4),
• a stronger decrease of emotional exhaustion (H5),
• a stronger decrease of perceived stress (H6).

We assume that all differences in changes occur immediately after the training and at 1-month follow-up.

Additional hypotheses: the training group reports a higher increase in general self-efficacy (AH1) and internal locus of control (AH2) and a higher decrease in external locus of control (AH3) than the waiting control group directly and 1 month after the training.

## Materials and Methods

### Schedule of the Training

One elementary component of teacher trainings in Germany are 1-day internal school training courses, which are generally held annually at most schools. As changes in behavior and experience are difficult to achieve through 1-day interventions, the common 1-day format was upgraded by some elements that increase the probability of sustainable changes and can be more easily integrated into teachers’ professional and private lives than additional full-day events.

**FIGURE 1:**
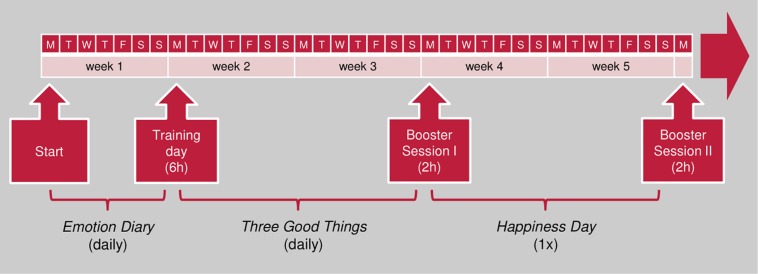
Training sequence.

Thus, a 5-week training phase with an entire training day and two 2-h booster sessions as well as small homework tasks before, during and after the training was designed (see Figure 1). The contents and exercises mentioned here are explained in more detail in the following two sections.

The training began with an e-mail-instructed *Emotion Diary* 1 week before the first meeting. Participants were requested to rate how intensely they experienced 20 given emotions and to note one situation with negative and one with positive emotions every evening. On the following 6-h training day, the experiences with the *Emotion Diary* were reflected upon and a large part of the contents (described in the following sections) was delivered. In the following 2 weeks, the participants carried out the cognitive PPI *Three Good Things*.

In the first 2-h booster session, participants’ experiences were reflected upon and the most important contents were reviewed. Additionally, mindfulness, savoring, and gratitude were introduced as further topics. The participants wrote a *Gratitude Letter* and thought about a personal *Savoring Experience*. The task for the next 2 weeks was to carry out a *Happiness Day* where participants choose to either make a *Gratitude Visit* or let the planned *Savoring Experience* become reality.

In the second and final booster session, the experiences with the *Happiness Day* were reflected upon and important contents from the previous sessions were repeated. The main objective of the last session was to ensure sustainability of the training effects. The participants first wrote a letter to themselves, which was to be opened a month later, reminding them of subjectively important topics, experiences, and intentions. The participants then worked out their personal happiness project with concrete plans for the near future.

An essential message within all sessions was the affirmation that everyone had much more internal control over their SWB than he or she was usually aware of. The total attendance time of the training was approximately 10 h, about 3 h had to be spent on the exercises at home (*Emotion Diary*, *Three Good Things*, and *Happiness Day*).

### Teaching Methods Used

The fundamental teaching methods included were psychoeducation in the sense of positive psychology, guided reflection, and homework between sessions.

#### Positive Psychoeducation

Psychoeducation in psychotherapy is to be understood as “systematic didactic-psychotherapeutic interventions […] which are suitable to inform patients and their relatives about the disorder and its treatment, to promote the understanding and the self-responsible handling of the disorder and to support them in coping with the disease” (Bäuml and Pitschel-Walz, 2003, p. 3, own translation). Accordingly, through psychoeducation in positive psychology, participants are to become experts for the conditions under which well-being evolves. By means of interactive information transfer they learn to purposefully influence the frequency and intensity of both positive and negative emotions, life satisfaction, stress perception, personal growth, and flourishing. This type of positive psychoeducation was implemented in the training in form of short lectures (20–40 min) on specific topic units, followed by a reflection break (see below).

#### Guided Reflection

Reflection breaks were used to anchor the contents taught and experienced. After every unit, participants had time to reflect on subjectively significant information and insights. The task in the reflection breaks was (1) to review the information received, (2) to write down one to three sentences of personal relevance, and (3) to exchange ideas and thoughts about the topic with one or two other participants. Finally, issues of interest were collected in the plenary session and questions clarified. Reflection stimulating exercises were carried out at several phases during the training. For example, participants should think about what activities reliably generate positive emotions for them, what they would like to do, if time and money did not matter, or in which situations less perfectionism would be desirable. In addition to anchoring the content, the aim was to question current habits and thereby encourage participants to engage in changes in attitudes and behavior. In both booster sessions, sufficient time was given to reflect on the experiences with homework in order to provide support in case of difficulties or to reinforce successes through feedback and encouragement from the peer-group and the trainer.

#### Homework

Exercise is an important mechanism to consolidate changes in behavior and experience. In his extensive second-order meta-analysis, Hattie (2008) reports an overall effect size of only *d* = 0.29 for homework on learning success for students, but points out that effects are higher if learners are older and more self-regulated and if the homework is about practicing or repeating of learned contents. Also homework takes place in addition to learning effects in sessions and therefore can be considered an important enrichment. Homework also plays a decisive role in cognitive behavioral therapy (CBT) and trainings based on it.

### Contents of the Training

The training starts with a focus on the concept of SWB as a scientific construct for the everyday term “happiness” (Diener and Scollon, 2014). Benefits of positive and negative emotions and life satisfaction are explained from evolutionary, neurobiological, and psychological perspectives. Favorable conditions for creating SWB are explained on the basis of the three basic psychological needs (competence, autonomy, and relatedness) of self-determination theory (Deci and Ryan, 2000). Information on the consequences of high SWB (see above) is used in particular to create personal relevance and motivation for the participants.

The *broaden-and-build theory of positive emotions* (Fredrickson, 1998, 2013, see above) plays an important role in the training. It is emphasized to the participants that increasing the personal frequency of PA empowers self-reinforcing upward spirals leading to build enduring personal resources and a more successful, pleasant, and meaningful life. By performing the PPIs described below, participants are animated to experience positive emotions more consciously, more frequently, and more intensely, thereby setting these upward spirals in motion.

To address the aim of decreasing the frequency of NA, neurobiological, psychological, and physiological conditions and consequences of NA are explained and strategies to regulate NA and stress (like change of cognitions, consciously distracting oneself, active coping and acceptance) are discussed (cf. Gross, 1998). Participants learn about the functions of NA and are thereby empowered to distinguish better between helpful and unhelpful NA. The most important learning objective in this regard is to realize that the frequency of occurrence of unhelpful emotions and even more their persistence is changeable.

Additionally, to reduce stress from time pressure, time management techniques are taught and tried out. In particular, the focus here is on setting priorities that promote well-being when choosing and designing tasks, saying “no” to additional assignments, and questioning perfectionist demands. It is emphasized that one has more influence on the selection and order of activities than one is aware of (cf. Claessens et al., 2007). Participants are encouraged to prioritize their own well-being (Catalino et al., 2014) and to invest the time gained through time and priority management in activities that promote SWB (for an overview on the interconnection between SWB and time see Mogilner et al., 2018).

Participants are made aware of how SWL develops and that one can deliberately change the personal perspectives of what deems relevant in life (Diener et al., 2018). Also, individual processes of social comparison are addressed. It is explained how these processes sometimes undermine a fair assessment of one’s successes and standing in life. Participants are encouraged to think about what might increase their SWL.

In regard to perception processes and their biases, the *negativity bias* – prominent in positive psychology – receives special attention. It describes the tendency that negative, potentially threatening stimuli have a greater effect on our experience and behavior than positive stimuli (Baumeister et al., 2001; Rozin and Royzman, 2001). This distortion toward an increased attention to negative environmental stimuli is explained to the participants. It is made clear that with conscious attention control, we can influence how many positive things we perceive in our environment (the perception of positive things is particularly practiced with the PPI *Three Good Things*).

For changes in individual evaluation processes, the training also addresses the topic of causal attribution (Weiner, 2000). People seek causal explanations for their action results. Those who ascribe their success to internal and stable causes promote their self-esteem and brighten their basic mood. The training emphasizes that the individual can gain control over evaluation processes and that it is possible to improve one’s own attributional style.

### Positive Psychological Interventions Used

As Lyubomirsky and Layous (2013) point out, not all interventions have the same effect on all people. Their person-activity-fit model stresses the importance of a fit between interventions and individuals. Also Schueller’s (2014) review indicates that fitting activities achieve better effects and are more likely to be maintained. The training takes this into account by selecting and designing exercises that leave as much room as possible for their execution. Additionally, we tried to keep the required effort for them as low as possible.

#### Emotion Diary

The *Emotion Diary* is a proprietary translation and adaptation of the modified Differential Emotion Scale (mDES) used by Fredrickson (2011) in her *broaden-and-build theory* studies. Participants were asked to rate the intensity of 10 positive and 10 negative emotions. In addition, each day they were asked to write down one situation that led to positive emotions and one that led to negative emotions. The *Emotion Diary* should be filled out in the week before the training day and was instructed by e-mail. The aim of the intervention was to activate the participants before the first meeting and to make them perceive the variability of their own emotions. The exercise took about 5 min per day to complete. The collected situations could be worked on during the training.

#### Three Good Things

In the PPI *Three Good Things*, each evening, the participants were asked to write down three good experiences of the day which had provided positive emotions and to indicate what they themselves had contributed to these experiences. The first part of the exercise aims to increase awareness of positive experiences and thus to improve the frequency of positive emotions. The second part deliberately deviates from the more general original instruction of Seligman et al. (2005) in order to explicitly train a favorable attributional style by ascribing positive experiences to internal causes. Seligman et al. (2005), as well as the replication studies by Mongrain and Anselmo-Matthews (2012) or Gander et al. (2013), were able to show significant improvements in well-being, which built up over a period of 3 months and were still measurable after 6 months. Participants were encouraged to complete the exercise for at least a few days. The exercise booklet contained pages for 14-day practice. Since Gander et al. (2013) found that a 2-week exercise application did not bring any benefit for well-being, participants were informed accordingly and offered possibilities for the variation of the exercise (e.g., to focus on certain domains like work or hobby or to write down three funny things or the like) in order to prevent boredom effects. Also it was emphasized that they should stop after some days if it felt more like a burdensome duty than an enrichment. This exercise took about 5 min per day to complete.

#### Letter of Gratitude

The *Letter of Gratitude* is another PPI from the studies mentioned above. In the first booster session, participants were asked to write a letter in which they explicitly thanked an important person in their life. One way to increase the positive effect of the exercise (Seligman et al., 2005) is to read the letter to the addressee personally (*Gratitude Visit*), which could be chosen as one option for the individual design of the *Happiness Day* (see below).

#### Savoring Experience

Bryant et al. (2011) assume that “savoring” makes a decisive contribution to experiencing and intensifying positive emotions. In the first booster session, the participants were encouraged to imagine being given 2 h of time, which could only be invested in a *Savoring Experience*. One of the ideas was to be planned in detail and was the second option for the *Happiness Day* activity.

#### Happiness Day

The given framework of this mostly self-designable PPI consisted of planning and carrying out an activity of about 2 h that would most likely evoke positive emotions. Therefore, participants could choose between the two PPIs mentioned above. The option of carrying out a *Gratitude Visit* was very rarely chosen in all groups (in total only about 5 of the participants opted for it). The opportunity to indulge in a *Savoring Experience* on the other hand, met with great enthusiasm. Examples of the activities carried out included a visit to the sauna, a campfire or musical experiences.

### Methods to Increase Sustainability

Various elements were integrated into the training to ensure greater sustainability. One measure was to extend the training time by instructing a first exercise (*Emotion Diary*) by e-mail already 1 week before the meeting. Also, the participants were encouraged to form so-called implementation intentions (Gollwitzer, 1999) in order to increase the probability that the “homework” to be done during the training (*Emotion Diary*, *Three Good Things*, and *Happiness Day*) would actually be carried out. These implementation intentions have the form of an “if, then” sentence and have already been successfully applied in many contexts. In the second booster session, the participants had the opportunity to record important information and insights from the training and to formulate good wishes for their future selves in a letter to themselves. The letter should be opened on a fixed date approximately 1 month after the training. The final exercise of the training was to design a personal happiness project for the future and to specify behavior, life mottos, and further implementation intentions, so that concrete action plans would be available after the training.

### Measures

In this study, we assessed the frequency of positive and negative emotions (SPANE), general life satisfaction (SWLS), flourishing (BIT), emotional exhaustion (MBI-EE), perceived stress (PSS), general self-efficacy (GSE), and locus of control (IE-4) as well as demographic data (year of birth and gender), and a code for anonymous assignment with an online-questionnaire. In addition, participants answered 10 self-constructed items on their subjective training success.

#### Frequency of Positive and Negative Experiences

The frequency of positive and negative emotions is part of the target construct of SWB and is assessed with the Scale of Positive And Negative Experiences (SPANE; Original: Diener et al., 2010; German version: Rahm et al., 2017). The instrument distinguishes between two subscales, each consisting of six items. Each subscale comprises three more general feelings (e.g., “pleasant” or “unpleasant”) and three more specific feelings (e.g., “joyful” or “sad”). Participants are asked how often they experienced the given emotion in the past 4 weeks and answer on a five-point scale from 1 (very rarely or never) to 5 (very often or always).

#### General Life Satisfaction

The general life satisfaction represents the cognitive component of SWB and is measured using the Satisfaction With Life Scale (SWLS; Original: Diener et al., 1985; German version: Glaesmer et al., 2011). The instrument consists of five items on a seven-point response scale from 1 (strongly disagree) to 7 (strongly agree). Example items are: “In most ways, my life is close to my ideal” or “If I could live my life over, I would change almost nothing.”

#### Flourishing

The construct of flourishing (or thriving) is considered to be a more comprehensive measurement of multidimensional well-being. The Brief Inventory of Thriving (BIT; Original: Su et al., 2014; German version: Hausler et al., 2017) used here is the unidimensional short version of the Comprehensive Inventory of Thriving (ibid.) and contains a total of 10 items on meaning, optimism, emotional state, flow, goal achievement, energy experience, and sense of belonging, which are evaluated on a five-point Likert scale from 1 (strongly disagree) to 5 (strongly agree). Example items are: “What I do in life is valuable and worthwhile” or “In most activities I do, I feel energized.”

#### Emotional Exhaustion

The Maslach Burnout Inventory (MBI; Original: Maslach et al., 1996; German version: Büssing and Perrar, 1992) measures the burnout syndrome on three subscales. In this study, we only used the subscale Emotional Exhaustion (MBI-EE), as the corresponding construct is considered to be the main symptom of burnout. Using five items (e.g., “I feel burned out from my work” or “Working all day is really a strain for me”), participants estimate the frequency of the experienced feeling on a seven-point scale from 0 (never) to 6 (every day).

#### Perceived Stress

The perceived stress was measured with the Perceived Stress Scale (PSS; Original: Cohen et al., 1983; German version: Klein et al., 2016). The instrument comprises 10 items on a five-point scale from 0 (never) to 4 (very often). Example items are: “In the last month, how often have you been upset because of something that happened unexpectedly?” or “In the last month, how often have you felt that you were unable to control the important things in your life?”

#### General Self-Efficacy

The general self-efficacy describes the confidence in mastering difficult situations and is measured by the General Self-Efficacy Scale (GSE; Original: Schwarzer and Jerusalem, 1999; English version: Schwarzer and Jerusalem, 1995). The scale contains 10 items that are to be rated on a five-point Likert scale from 1 (not at all true) to 4 (exactly true). Example items are: “I can remain calm when facing difficulties because I can rely on my coping abilities” or “I can usually handle whatever comes my way.”

#### Internal–External Locus of Control

Control beliefs were recorded using the German scale Internale-Externale-Kontrollüberzeugungen-4 (IE-4; Original: Kovaleva et al., 2014; English version: Kovaleva, 2012). The scale comprises two subscales, one for the internal locus of control and one for the external locus of control, each consisting of two items. All four items are rated on a five-point Likert scale from 1 (doesn’t apply at all) to 5 (applies completely). Example items are: “If I work hard, I will succeed” (internal) or “Fate often gets in the way of my plans” (external).

#### Subjective Training Success

For the assessment of the subjective training success we constructed 10 additional items which should be answered on a seven-point Likert scale from 1 (not applicable at all) to 7 (very applicable). Example items are: “I benefited from the training” or “I can perceive good things in my environment better than before the training.”

### Sample

The training was offered in three institutions. In two German schools, a grammar school (Gymnasium) and a vocational school, participants were recruited via short presentations held at the teachers’ council that briefly described aims, contents, and schedule of the training. Interested teachers could voluntarily participate in the training in their working hours. As the third institution, the institute of educational psychology offered the training to staff of the Technische Universität Braunschweig (recruited via the department of vocational training) and to public (recruited via a newsletter for people interested in positive psychology).

For each of the three institutions (grammar school, vocational school, and university) the training was delivered twice. The participants of the first training round acted as the training group. The second training round was necessary in order to set up a waiting control group with similarly interested participants – effects of the second training were therefore not included in the data analyses. Participants were assigned to the groups by taking individual time restrictions into account to realize a sufficiently large sample. Table 1 shows the distribution of the *N* = 89 participants among the groups and institutions. The participants were between 24 and 67 years old (*M* = 46.2; *SD* = 11.4), 74.2% were female.

**TABLE 1:** Distribution of the participants among groups and institutions.

***N* = 89**	**Training group**	**Control group**
Grammar school	15	20
Vocational school	13	13
University	14	14
**Total**	**42**	**47**

### Procedure

**FIGURE 2:**
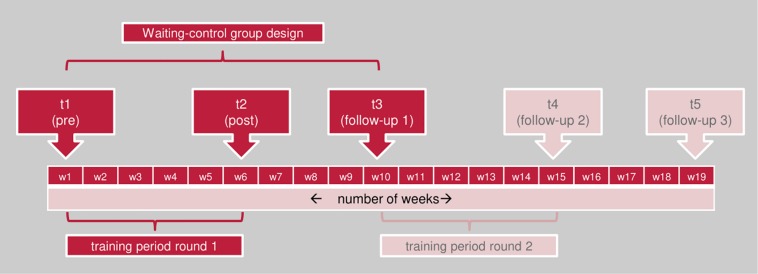
Study design and measurement times.

The training was delivered twice in each institution by the first author. Figure 2 shows the sequence of the measurement points in accordance to the training periods for each institution. All participants (training and control group) from one institution (grammar school, vocational school, or university) answered the questionnaires at the same time, while the exact training dates and measurement points differed between the institutions. The first training period for the grammar school started in April, for the vocational school in Mid-August and for the university at the end of August, all in 2017.

At measurement time point 1 (t1: pre), all participants from the respective institution received an e-mail asking them to participate in an online survey. For the participants of the training group of this institution, this e-mail also contained the instruction for the first exercise (*Emotion Diary*). One week after t1, the training group of this institution had their training day, 2 weeks later booster session 1, and another 2 weeks later booster session 2. One day after the last booster session, all participants (training and control group) from this institution received an e-mail asking them to take the second online survey at t2 (post). The waiting control group had to be trained in the same school year. Due to organizational reasons, the second round of the training period (for the waiting control groups) had to start at different times in the different institutions. Therefore, the follow-up measurement t3 took place with different time intervals (29 days for the vocational school, 36 for the university course and 70 for the grammar school) after t2.

To investigate long term effects, participants were asked to complete two more questionnaires. The second follow-up (t4) took place 9 weeks (vocational school) to 15 weeks (grammar school) after the end of training, the third follow-up (t5) took place 19 to 25 weeks after the end of training. As the waiting control group had already received the training at t4 and t5, the analyses of long term effects could only be conducted without a control group.

For each measurement point, depending on the response rate, one or two reminder e-mail(s) were sent a few days after the first request. The online-surveys were created with the software Questback and answered anonymously within 1 week after the according request per e-mail. All participants were informed in detail about the voluntary nature of their participation, the measures taken to maintain anonymity, and the purpose of the study and gave their written informed consent for further data processing. The study was approved by the Ethics Committee of the Faculty of Life Sciences, Technische Universität Braunschweig (FV-2019-07).

### Data Analysis

First, a *t*-test was used to check whether training and control group differed in their initial values. The changes from t1 to the other measurement points within the groups were tested with *t*-tests for dependent samples. Cohen’s *d* was calculated as an effect measure using the standard deviation of the mean differences in accordance to Field (2014). The differences in changes between groups were tested by planned contrasts. For each of the scales used, the respective mean differences between the measurement points (post: t2 minus t1; follow-up: t3 minus t1) were compared between training and control group using *t*-tests. Cohen’s *d* was calculated using the pooled standard deviation at t1 and the bias correction suggested by Morris (2008). In case of specific hypotheses, planned contrasts provide stronger statistical power than ANOVA (Furr, 2008). Power analyses indicate that with the given sample size and α = 0.05, effect sizes of *d* ≥ 0.50 could be detected with 1 – β = 0.80.

To compare the same participants at all measurement points, only cases with complete data at t1, t2, and t3 were included in the analyses. Initially 106 participants answered the survey at t1. 17 participants (9 from the training and 8 from the control group) did not take part in t2 or t3 and were therefore excluded from analyses, leading to a total sample of *N* = 89 participants. A *t*-test of mean differences between the *N* = 89 included and the *n* = 17 excluded participants showed no significant differences on five of the six target variables – only the SWLS indicated a significantly higher mean for the included cases. As Questback enforced complete answers to the questionnaires, there are no missing values in single items of the questionnaires.

To test long-term effects, changes from t1 to t4 and from t1 to t5 in the training group (*n* = 42) were tested for significance using *t*-tests. As some of the participants did not take part in these measurement points, these analyses had to be carried out with reduced sample sizes (t4: *n* = 30; t5: *n* = 35). Again Cohens *d* was calculated. (The results of these analyses, however, are to be interpreted cautiously as a control group could not be realized here.) All calculations were carried out with SPSS 25.

## Results

Table 2 shows the mean values, standard deviations, and internal consistencies of the measures used at t1 for all participants. With the exception of the subscale for external locus of control (α = 0.58), all scales achieved at least good values of internal consistencies (α > 0.80). The initial values (t1) did not show significant mean differences between the training and the control group on any scale.

**TABLE 2:** Scale characteristics (pre).

**Construct**	**Scale**	**Number of items**	**Range**	***M***	***SD***	**Cronbach’s α**
Frequency of positive emotions	SPANE-P	6	6–30	20.90	4.11	0.89
Frequency of negative emotions	SPANE-N	6	6–30	14.71	4.25	0.81
General satisfaction with life	SWLS	5	5–35	25.26	5.91	0.90
Flourishing	BIT	10	10–50	37.36	5.41	0.84
Emotional exhaustion	MBI-EE	5	0–30	17.21	4.93	0.87
Perceived stress	PSS	10	0–40	26.17	6.01	0.85
General self-efficacy	GSE	10	10–40	28.84	4.67	0.91
Internal locus of control	IE-4-IK	2	2–10	7.63	1.51	0.82
External locus of control	IE-4-EK	2	2–10	4.81	1.75	0.58

*M = mean; SD = standard deviation; *N* = 89.*

Table 3 shows the results of the *t*-tests for mean differences between the measurement points t1 (pre) and t2 (post) within-groups. With the exception of the variables *general self-efficacy* and *internal* and *external locus of control*, all variables in the training group (*n* = 42) improved significantly from t1 to t2. The waiting control group (*n* = 47) showed no significant changes from t1 to t2, except for the decrease of the *frequency of positive emotions*. Analyses of the interaction contrasts are shown in Table 4. The between-group effects show a significantly higher increase in *frequency of positive emotions* (H1), *life satisfaction* (H2), and *flourishing* (H3) in the training group than in the control group (interaction effects *d* = 0.65, *d* = 0.40, and *d* = 0.43) and a significantly stronger decrease in *frequency of negative emotions* (H4), *emotional exhaustion* (H5), and *perceived stress* (H6) with interaction effects of *d* = 0.53, *d* = 0.42, and *d* = 0.49. *Self-efficacy* and *internal* and *external locus of control* were not significantly influenced.

**TABLE 3:** Within-group training effects 1 day after training (pre–post).

	**Training group (*n* = 42)**							**Waiting control group (*n* = 47)**						
	***M1***	***SD1***	***M2***	***SD2***	***T***	***df***	***d***	***M1***	***SD1***	***M2***	***SD2***	***T***	***df***	***d***
Positive emotions	21.21	4.08	23.02	3.95	–2.684	41	0.41^∗^	20.62	4.16	19.74	4.05	2.111	46	−0.31^∗^
Negative emotions	14.86	4.48	12.98	4.33	2.966	41	–0.46^∗∗^	14.57	4.07	14.98	3.81	–0.796	46	0.12
Satisfaction with life	26.26	5.79	27.88	4.79	–2.771	41	0.43^∗∗^	24.36	5.93	23.60	5.52	1.936	46	–0.28
Flourishing	37.71	5.51	39.81	4.85	–2.870	41	0.44^∗∗^	37.04	5.36	36.77	5.34	0.717	46	–0.10
Emotional exhaustion	17.64	5.57	16.19	5.59	2.324	41	−0.36^∗^	16.83	4.31	17.49	4.47	–1.245	46	0.18
Perceived stress	26.00	6.91	23.60	6.18	2.763	41	–0.43^∗∗^	26.32	5.15	26.91	5.91	–0.807	46	0.12
General self-efficacy	29.36	5.36	30.31	3.85	–1.831	41	0.28	28.38	3.96	28.57	4.17	–0.559	46	0.08
Internal locus of control	3.93	0.72	4.13	0.63	–1.831	41	0.28	3.71	0.78	3.77	0.61	–0.759	46	0.11
External locus of control	2.32	0.94	2.24	0.77	0.774	41	–0.12	2.48	0.77	2.50	0.76	–0.244	46	0.04

*Mx = mean value at measurement time X; SDx = standard deviation at measurement time X; d = Cohen’s d; ^∗^*p* < 0.05; ^∗∗^*p* < 0.01.*

**TABLE 4:** Between-group differences of within-group differences between t2 and t1 (pre–post).

	**training (*n* = 42)**		**control (*n* = 47)**				
	***M*_ߡ__post_**	***SD*_ߡ__post_**	***M*_ߡ__post_**	***SD*_ߡ__post_**	***T***	***df***	***d***
Positive emotions	1.81	4.37	–0.87	2.83	3.392	68.932	0.65^∗∗^
Negative emotions	–1.88	4.11	0.40	3.48	–2.840	87	–0.53^∗∗^
Satisfaction with life	1.62	3.79	–0.77	2.71	3.442	87	0.40^∗∗^
Flourishing	2.10	4.73	–0.28	2.64	2.960	87	0.43^∗∗^
Emotional exhaustion	–1.45	4.05	0.66	3.63	–2.594	87	−0.42^∗^
Perceived stress	–2.40	5.64	0.60	5.06	–2.646	87	–0.49^∗∗^
General self-efficacy	0.95	3.37	0.19	2.35	1.246	87	0.16
Internal locus of control	0.20	0.72	0.05	0.48	1.140	70.449	0.20
External locus of control	–0.08	0.70	0.02	0.60	–0.761	87	–0.12

*M_Δ__*post*_ = mean difference between measurement points t2 and t1; SD_Δ__*post*_ = standard deviation of the difference; T = T-Value; df = degrees of freedom in *t*-test or Welch-test; d = Cohen’s *d; ^∗^p* < 0.05; *^∗∗^p* < 0.01.*

Table 5 shows the results of the *t*-tests for mean differences between the measurement points t1 (pre) and t3 (follow-up1) within groups. With the exception of the variable *external locus of control*, all variables in the training group (*n* = 42) improved significantly from t1 to t3. In the waiting control group (*n* = 47), we found a significant improvement in *general self-efficacy*. Analyses of the interaction contrasts are shown in Table 6. The between-group differences of within-group changes show a significantly higher increase in *frequency of positive emotions* (H1), *life satisfaction* (H2), and *flourishing* (H3) in the training group than in the control group (interaction effects *d* = 0.44, *d* = 0.31, and *d* = 0.32) and a significantly stronger decrease in *frequency of negative emotions* (H4), *emotional exhaustion* (H5), and *perceived stress* (H6) with interaction effects of *d* = 0.69, *d* = 0.47, and *d* = 0.51). Additional to the results at t2, an interaction effect on *internal locus of control* could be found (*d* = 0.38). *External locus of control* and *general self-efficacy* were not significantly influenced.

**TABLE 5:** Within-group training effects 1 month after training (pre – follow-up1).

	**Training group (*n* = 42)**							**Waiting control group (*n* = 47)**						
	***M1***	***SD1***	***M3***	***SD3***	***T***	***df***	***d***	***M1***	***SD1***	***M3***	***SD3***	***T***	***df***	***d***
Positive emotions	21.21	4.08	23.55	3.42	–3.391	41	0.52^∗∗^	20.62	4.16	21.13	3.55	–0.913	46	0.13
Negative emotions	14.86	4.48	11.76	3.60	5.202	41	–0.80^∗∗^	14.57	4.07	14.47	3.92	0.205	46	–0.03
Satisfaction with life	26.26	5.79	28.19	4.68	–3.104	41	0.48^∗∗^	24.36	5.93	24.45	4.74	–0.186	46	0.03
Flourishing	37.71	5.51	39.95	4.79	–3.755	41	0.58^∗∗^	37.04	5.36	37.53	4.92	–0.990	46	0.14
Emotional exhaustion	17.64	5.57	15.29	5.61	4.226	41	–0.65^∗∗^	16.83	4.31	16.81	3.76	0.040	46	–0.01
Perceived stress	26.00	6.91	21.79	5.40	5.076	41	–0.78^∗∗^	26.32	5.15	25.23	5.98	1.141	46	–0.17
General self-efficacy	29.36	5.36	31.43	4.36	–3.555	41	0.55^∗∗^	28.38	3.96	29.32	4.01	–2.415	46	0.35^∗^
Internal locus of control	3.93	0.72	4.24	0.67	–3.164	41	0.49^∗∗^	3.71	0.78	3.73	0.75	–0.313	46	0.05
External locus of control	2.32	0.94	2.17	0.80	1.410	41	–0.22	2.48	0.77	2.50	0.90	–0.182	46	0.03

*Mx = mean value at measurement time X; SDx = standard deviation at measurement time X; d = Cohen’s d; ^∗^*p* < 0.05; ^∗∗^*p* < 0.01.*

**TABLE 6:** Between-group differences of within-group differences between t3 and t1 (pre – follow-up1).

	**Training (*n* = 42)**		**Control (*n* = 47)**				
	***M_ߡ__fu1_***	***SD_ߡ__fu1_***	***M_ߡ__fu1_***	***SD_ߡ__fu1_***	***T***	***df***	***d***
Positive emotions	2.33	4.46	0.51	3.83	2.073	87	0.44^∗^
Negative emotions	–3.10	3.86	–0.11	3.56	–3.799	87	–0.69^∗∗^
Satisfaction with life	1.93	4.03	0.09	3.13	2.424	87	0.31^∗^
Flourishing	2.24	3.86	0.49	3.39	2.276	87	0.32^∗^
Emotional exhaustion	–2.36	3.61	–0.02	3.63	–3.036	87	–0.47^∗∗^
Perceived stress	–4.21	5.38	–1.09	6.52	–2.453	87	−0.51^∗^
General self-efficacy	2.07	3.78	0.94	2.66	1.654	87	0.24
Internal locus of control	0.31	0.63	0.02	0.47	2.462	87	0.38^∗^
External locus of control	–0.15	0.71	0.02	0.80	–1.091	87	–0.20

*M_Δ__*fu1*_ = mean difference between measurement points t3 and t1; SD_Δ__*fu1*_ = standard deviation of the difference; T = T-Value; df = degrees of freedom in *t*-test or Welch-test; d = Cohen’s d; ^∗^*p* < 0.05; ^∗∗^*p* < 0.01.*

In summary, all main hypotheses (H1 to H6) were confirmed, the additional hypotheses only for *internal locus of control* and only at t3 (AH2).

Outside the waiting control group design, two additional measurement points were implemented for the training group round 1. The first was about 3 months (t4: 9–15 weeks) after the training and the second about 5 months (t5: 19–25 weeks). As can be seen in Table 7, most instruments also revealed a significant improvement compared to t1. The improvements in the *frequency of positive and negative emotions* were not significant at t5. It is important to emphasize that no comparative data from control groups are available for this period, as the waiting control group had already received the training at this time.

**TABLE 7:** Long-term changes in the training group – without control group.

	**9–15 weeks after training(*n* = *30)***							**19–25 weeks after training(*n* = *35)***						
	***M1***	***SD1***	***M4***	***SD4***	***T***	***df***	***d***	***M1***	***SD1***	***M5***	***SD5***	***T***	***df***	***d***
Positive emotions	20.73	4.14	23.40	3.64	–3.281	29	0.60^∗∗^	20.86	3.81	22.74	5.02	–1.834	34	0.31
Negative emotions	15.37	4.48	12.60	3.89	3.966	29	–0.72^∗∗^	14.86	4.53	13.34	5.14	1.376	34	–0.23
Satisfaction with life	25.57	6.37	28.10	4.90	–2.763	29	0.50^∗∗^	26.11	6.06	27.77	5.11	–2.307	34	0.39^∗^
Flourishing	37.27	5.92	40.63	4.89	–3.822	29	0.70^∗∗^	37.83	5.67	40.29	4.87	–3.225	34	0.55^∗∗^
Emotional exhaustion	18.27	5.58	15.67	6.02	4.390	29	–0.80^∗∗^	17.34	5.80	15.09	5.87	3.115	34	–0.53^∗∗^
Perceived stress	27.07	6.87	22.73	5.79	3.881	29	–0.71^∗∗^	26.06	6.98	22.00	6.31	3.835	34	–0.65^∗∗^
General self-efficacy	29.00	5.30	30.80	4.29	–2.250	29	0.41^∗^	29.46	5.12	31.74	4.16	–3.139	34	0.53^∗∗^
Internal locus of control	3.83	0.80	4.03	0.61	–1.682	29	0.31	3.84	0.73	4.21	0.53	–3.465	34	0.59^∗∗^
External locus of control	2.32	1.00	2.23	0.83	0.634	29	–0.12	2.33	0.93	2.10	0.64	1.992	34	–0.34

*Mx = mean at measurement time X; SDx = standard deviation at measurement time X; d = Cohen’s d; ^∗^*p* < 0.05; ^∗∗^*p* < 0.01.*

In addition to the psychological constructs mentioned, participants were asked to report their subjective training success. Table 8 shows the items in wording with mean values and standard deviations at t5. In particular, the participants reported a high personal benefit (*M* = 5.68, *SD* = 1.04 on a seven-point Likert scale, where 7 indicates the highest agreement) and a subjective increase in knowledge (*M* = 5.44; *SD* = 1.08). The values of all items are above the average value of the scale (4.00).

**TABLE 8:** Subjective training success 19–25 weeks after training.

**Item**	***M***	***SD***
I benefited from the training.	5.68	1.04
I can perceive good things in my environment better than before the training.	5.44	1.26
I experience more positive emotions than before the training.	5.12	1.30
I focus more on the present moment than before the training.	5.24	1.23
I can handle negative emotions better than before the training.	4.71	1.09
I enjoy now more consciously than before the training.	5.35	1.28
I feel more grateful than I did before the training.	5.21	1.15
I am more often friendly and helpful than before the training.	4.32	1.27
I learned a lot for myself in the training.	5.44	1.08
I have become more satisfied through the training.	5.12	1.23

**n* = 34; M = mean; SD = standard deviation; range: 1 (strongly disagree) – 7 (strongly agree).*

At each measurement point, the training participants also had the opportunity to leave anonymous comments on particular strengths and improvement ideas, which was frequently used. The positive comments often related to gratitude for the training and for the impulses for personal life improvement or referred to concrete personal successes attributed to the training. A number of participants also expressed that some or most of the contents had already been known before, but that it was good to be assured of “doing it right.” Suggestions for improvement were, among other things, often related to individual exercises (e.g., the *Letter of Gratitude* was viewed critically) or the timing (more time for or between booster sessions). Quite often the wish for an additional booster session after about half a year was expressed. All in all, the open statements showed a positive picture and a high level of satisfaction with the training.

## Discussion

In general, it could be shown that the developed training was able to meet the goal of promoting SWB. Immediately after the training (t2: post), the participants in the training group showed significantly stronger increases in *frequency of positive emotions, life satisfaction*, and *flourishing* than the participants in the control group and a significantly stronger decrease in *frequency of negative emotions*, *emotional exhaustion*, and *perceived stress*, accordingly. The effects persisted about 1 month (t3: follow-up 1) after the end of the training. Thus, all main hypotheses could be confirmed. Therefore, the present study adds another effective multi-component intervention to the field of positive psychology. However, because of the methodological limitations (see below), further studies should be carried out to validate the results.

Up to date, there are three meta-analyses on the effectiveness on (multi-component) PPIs (Sin and Lyubomirsky, 2009; Bolier et al., 2013; Hendricks et al., 2019), revealing small to medium effect sizes for the enhancement of well-being and the reduction of depressive symptoms. Results of the recent study are therefore in line with other findings. In these meta-analyses, different measures for well-being were used. It is worth mentioning that the present study seems to be one of few taking the measurement of NA into account which seems surprising, as the construction of SWB with its three composites (SWL, PA, and NA) is quite common (Diener, 1984; Pavot and Diener, 2013) and most of the studies included in these meta-analyses claim to increase SWB. We deem it remarkable that we found the strongest effects at t3 in the reduction of the frequency of negative emotions, although this was not trained directly in the exercises between the training day and the booster sessions. The influence on this variable thus seems to be achieved mainly through positive psychoeducation and general reflections or as side effects of the PPIs. Therefore, we recommend including the measurement of NA in future intervention studies (see below).

A main learning objective of the present training in total and especially of the PPI *Three Good Things* was to strengthen the belief of the participants to have more influence on their SWB than they are usually aware of and to motivate them to make use of this influence by changing behavior. Therefore, our additional hypotheses expected increases in general self-efficacy (AH1) and internal locus of control (AH2) and decreases in external locus of control (AH3). This was only partly confirmed by a significant increase in internal locus of control and only between t1 and t3 (AH2). In regard of general self-efficacy, it seems plausible that the training has no significant effect on this broad assessment of beliefs about mastery or problem-solving expectations and more specific self-efficacy constructs should be considered (see below). For locus of control it might also be possible that the short scales used in the present study with only two items for each subscale were not suitable to capture all relevant aspects of the constructs. Additionally, the subscale for external locus of control only revealed a Cronbach’s alpha of 0.58. Therefore, we recommend using a more comprehensive scale in future studies.

Compared to the first time of measurement (t1: pre), significant improvements for the training groups were also observed after approximately two and a half (t4: follow-up 2) and approximately 5 months (t5: follow-up 3) respectively – however, due to the lack of a control group, these results could also be attributed to other circumstances so that they can only be interpreted cautiously as a first indication of long-term effects. The frequency of positive and negative emotions did not significantly change from t1 to the follow-up after approximately 5 months (t5). Since there was no control group at t5, it cannot be tested whether the training possibly prevented a decline in emotions caused by external influences such as more stressful phases at school or if this result is due to hedonic adaptation processes or due to the small sample size.

In addition to its overall effectiveness, the training has the advantage of a manageable amount of time, which could facilitate (active) participation: the personal contact time was only 10 h in total, divided into one training day and two booster sessions, and the self-structured exercises lasted only about 3 h in total over a period of 5 weeks. The feedback received during the training, the results of the subjectively assessed effects of the training, and the open comments showed that the offer was well-received by the target group. The integration of coping strategies for aversive emotions and time management techniques probably also contributed to this. Both contents are known to be important skills for work performance and health of teachers (Skaalvik and Skaalvik, 2018) but have rarely been considered in multi-component interventions claiming to increase SWB.

### Limitations and Further Research

Due to the field research approach, this study has two obvious limitations: (1) For organizational reasons in the schools, the follow-up measurement could not be carried out in all three groups after exactly 30 days so that there was a notable difference in the amount of time between the end of the training and the first follow-up measurement (t3) for the three institutions, with the grammar school being assessed more than 1 month later (70 days in comparison to 29 and 36 days). (2) The first two training groups consisted of teachers from one school each, while the third group also included some teachers from different schools but mostly members of other professions. In our opinion, however, the fact that the training has proven effective even under these suboptimal conditions indicates the robustness of the effects. Nevertheless, in subsequent studies these restrictions should be avoided.

As mentioned above, 17 participants who did not take part in all three measurement points were excluded from the analyses. These excluded cases revealed a significantly lower life satisfaction at t1. Therefore, it seems that less contented people might also be less motivated to complete the follow-up questionnaires. To avoid biases in this regard, one could consider working with incentives in future studies.

Another issue are the shortcomings of the evaluation design. As Hendricks et al. (2019) found an effect of the study quality in their meta-analysis (expressed, e.g., in randomization and placebo-control) showing lower effects in high-quality studies, it may be that the effects of the present study are overestimated. In a larger project with more resources for recruiting schools and teachers, it should be possible to secure a larger sample size which would be especially favorable for two different approaches: (1) A cross-school training with randomized assignment of the participants to the groups including an active alternative-treatment-control group would avoid biases concerning the selection procedure (which was based on available time of the participants in this study) and could ensure that changes are due to the training and not due to social interaction or other confounded variables. Additionally, as more research about the precise mechanisms of PPIs and multi-component interventions is still needed (Mongrain and Anselmo-Matthews, 2012; Chow, 2018), it would be even more valuable to also carry out a component evaluation to check which individual components are responsible for the effects. Therefore, one could develop several independent training modules (e.g., positive emotions, emotion regulation, or time management) and apply them in different order to several groups to find out which modules affect which facets of well-being. (2) In-school trainings with several participating schools would allow to investigate the influence of school-specific variables such as openness to change, peer support, experience of stress, or general school climate on the sustainability of training successes using multi-level analyses. For example, Ebersold et al. (2019) were able to show that teachers’ well-being was significantly influenced by the perceived autonomy support by their principals, mediated by satisfaction of the basic psychological needs for autonomy, competence, and relatedness (Deci and Ryan, 2000). It can be assumed that effects of well-being interventions will last longer in a supportive environment.

As the research on the *broaden-and-build theory* revealed favorable outcomes of increasing the frequency of PA, such as higher resilience, social connection, and functioning at optimal levels (Fredrickson, 2013), one can hope that the present training will also contribute to build such enduring resources not targeted directly. This, of course, needs to be examined in additional studies preferably with long-term follow-ups 6 or more months after intervention. Regarding the inclusion of interventions to decrease the frequency of NA, it would also be of interest if such combined interventions targeting all components of SWB could be even more effective than focusing PPIs only.

In addition to the constructs used here for evaluation, the construct of self-efficacy seems especially promising, as it is associated both with well-being (e.g., Luszczynska et al., 2005) and with intention building and behavior (e.g., physical activity, Rovniak et al., 2002). Since general self-efficacy was not affected in this study, domain-specific variants should be used in further investigations. In the school context, Schmitz and Schwarzer (2000) were already able to show correlations between teacher-specific self-efficacy and job satisfaction or burn-out. Zee and Koomen (2016) synthesized 165 articles on teacher self-efficacy (TSE) from over 40 years of research concluding: “Results suggest that TSE shows positive links with students’ academic adjustment, patterns of teacher behavior and practices related to classroom quality, and factors underlying teachers’ psychological well-being, including personal accomplishment, job satisfaction, and commitment” (p. 981). Furthermore, in the field of positive psychology, a scale for measuring well-being-related self-efficacy with seven factors (positive focus, emotion regulation, engagement, connectedness, meaning, goal achievement, and time sovereignty) is currently being constructed and validated (Rahm et al., in preparation).

In order to better classify results from interventions such as well-being trainings, but also applications of short PPIs or even more complex changes such as school development processes, it would be helpful to obtain more precise information about the fluctuations in well-being over the course of the (school) year. Findings about such general variations might also contribute to the explanation of effects of PPIs and multi-component interventions. In addition, knowledge about periods regarding well-being or ill-being, such as more relaxed or more stressful times, could be used to determine the right timing for well-being interventions. Furthermore, findings in this area could also make valuable contributions to the current discussion on hedonic adaptation and set point theory (Brown and Rohrer, 2019; Luhmann and Intelisano, 2018).

Another promising field for further research is the influence of improved teacher well-being on students. What happens to school climate and class climate, what happens to pedagogical relationships, and what happens to the well-being and academic performance of the students if the teaching staff is continuously trained and supported in the sense of positive psychology?

## Conclusion

In summary, it can be concluded that the training was well-received by the participants and increased their SWB for at least 1 month after the training. Due to the low organizational and time expenditure, it can be easily integrated into the training systems for teachers. In the context of the growing field of positive education, the training could prove to be a motivating starting point for school development processes to promote well-being and flourishing for teachers and students if the results found here can also be replicated in methodologically stricter research designs.

## Data Availability Statement

The datasets generated for this study are available on request to the corresponding author.

## Ethics Statement

The studies involving human participants were reviewed and approved by Ethical committee of the Faculty 2, Technische Universität Braunschweig (FV-2019-07). The patients/participants provided their written informed consent to participate in this study.

## Author Contributions

TR developed and delivered the training, organized the data collection, prepared the data set for the analyses, conducted the analyses, and wrote the first draft of the manuscript. EH scientifically supervised each step of the study, contributed to the development and the evaluation design of the training and was substantially involved in the final version of the manuscript.

## Conflict of Interest

The authors declare that the research was conducted in the absence of any commercial or financial relationships that could be construed as a potential conflict of interest.
